# Untethered Magnetic Soft Robot with Ultra‐Flexible Wirelessly Rechargeable Micro‐Supercapacitor as an Onboard Power Source

**DOI:** 10.1002/advs.202303918

**Published:** 2023-08-06

**Authors:** Swapnil Shital Nardekar, Sang‐Jae Kim

**Affiliations:** ^1^ Nanomaterials & System Lab Major of Mechatronics Engineering Faculty of Applied Energy System Jeju National University Jeju 63243 Republic of Korea; ^2^ Nanomaterials & System Lab Major of Mechanical System Engineering College of Engineering Jeju National University Jeju 63243 Republic of Korea; ^3^ Research Institute of New Energy Industry (RINEI) Jeju National University Jeju 63243 Republic of Korea

**Keywords:** integrated energy devices, magnetic soft‐robot, micro‐supercapacitor, wireless charging, onboard power source

## Abstract

Soft robotics has developed rapidly in recent years as an emergent research topic, offering new avenues for various industrial and biomedical settings. Despite these advancements, its applicability is limited to locomotion and actuation due to the lack of an adequate charge storage system that can support the robot's sensory system in challenging conditions. Herein, an ultra‐flexible, lightweight (≈50 milligrams), and wirelessly rechargeable micro‐supercapacitor as an onboard power source for miniaturized soft robots, capable of powering a range of sensory is proposed. The simple and scalable direct laser combustion technique is utilized to fabricate the robust graphene‐like carbon micro‐supercapacitor (GLC‐MSC) electrode. The GLC‐MSC demonstrates superior areal capacitance (8.76 mF cm^−2^), and maintains its original capacitance even under extreme actuation frequency (1–30 Hz). As proof of conceptthe authors fabricate a fully integrated magnetic‐soft robot that shows outstanding locomotion aptitude and charged wirelessly (up to 2.4 V within 25s), making it an ideal onboard power source for soft robotics.

## Graphical abstract

Developing adequate power sources is always the most challenging aspect of robotics research. In this perspective, an ultra‐flexible, lightweight, and wirelessly rechargeable micro‐supercapacitor (MSC) as an onboard power source for next‐generation autonomous soft robots is proposed. The fabricated MSCs can empower onboard sensory systems, while maintaining energy and power ratings in challenging conditions, tackling a pivotal challenge in soft robotics research.

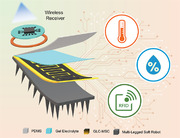

## 1 Introduction

Miniature soft robots that can be controlled by external stimuli such as thermal, photonic, pneumatic, hydraulic, electrical, and magnetic systems hold great importance for future biomedical, space, defense, and industrial applications.^[^ ^1^, ^2^, ^3^ ^]^ Distinct from conventional rigid systems, they offer many advantages such as joint‐less design, low mechanical stiffness, easy adaption to the outer environment, compact design, lightweight, and enabling low‐cost manufacturing process.^[^ ^4^, ^5^ ^]^ Researchers have recently succeeded in developing several small‐scale robots in various sizes and structures to execute sophisticated duties such as navigating across complicated territories, carrying heavy things, climbing over barriers, flying in air, swimming in a liquid environment, and so on.^[^ ^6^, ^7^, ^8^, ^9^, ^10^ ^]^ Although these strategies pave an important foundation in advancing soft‐robotics applications, their operations are limited to locomotion/actuation.^[^ ^11^, ^12^, ^13^ ^]^ Since being a soft multifunctional robot, which is equipped with multimodule sensory and communications modules for the real‐time data acquisition is equally essential rather than only actuation.^[^ ^3^, ^10^, ^14^ ^]^ In this regard, many efforts have been made to develop autonomous soft robots integrated with multisensory system that can sense temperature, strain, optical, humidity, bending, and other things.^[^ ^14^, ^15^, ^16^ ^]^ However, for the active operation of this sensory and communication hardware, designing flexible and portable power sources is always a big challenge due to the limited space and high weight of the power sources.^[^ ^3^, ^14^, ^17^ ^]^ Only a few attempts with small‐scale battery packs as a primary power source have been made in the last decade.^[^ ^18^, ^19^, ^20^ ^]^ However, these sources are constrained by several drawbacks, including their large weight, lack of flexibility, flammability, and high cost.^[^ ^21^, ^22^, ^23^ ^]^ Recently, supercapacitors, also known as electrochemical capacitors, have been regarded as efficient and promising electrochemical energy storage (EES) due to their advantages of rapid charging rate (in a few seconds), long cycle life, high power density, environmental friendliness, and economic efficiency.^[^ ^23^, ^24^, ^25^ ^]^ A flexible and compact EES device (such as a planner or micro‐supercapacitor) in particular reduces component sizes and power consumption in miniaturized electronic circuits, thereby filling a gap in meeting the energy and power supply requirements of micro‐sensory and can be directly integrated into soft robots.^[^ ^25^, ^26^, ^27^ ^]^

To address these limitations and as a proof of concept, we introduce the first approach for fabricating ultra‐flexible and lightweight, wirelessly rechargeable micro‐supercapacitors as an onboard power source that could allow the micro‐sensory system to operate continuously. Our fully integrated robot demonstrates superior functionalities such as excellent locomotion ability without losing its degrees of freedom. This work greatly expands the applicability of MSCs as a new onboard power source in the field of soft robotics.

## 2 Results and Discussion

**Figure 1:**
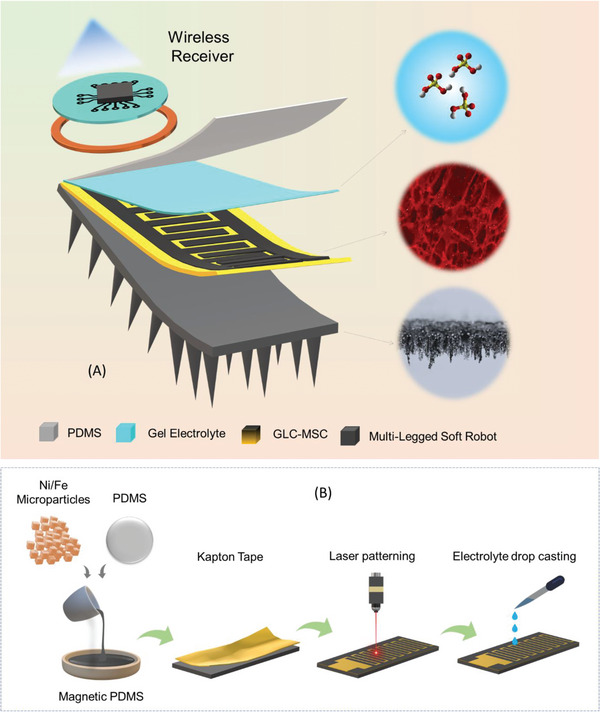
Fabrication of magnetic soft‐robot and GLC‐MSC. A) Schematic of magnetic soft‐robot with onboard GLC‐MSC and wireless charging system. B) Schematic representation for the fabrication of magnetic soft‐robot/actuator and GLC‐MSC.

The schematic illustration for the fabrication of the magnetic soft actuator/robot and laser‐combusted graphene‐like carbon micro‐supercapacitor (GLC‐MSC) is visualized in **Figure** **1**. The direct laser combustion technique was utilized to fabricate the interdigitated GLC‐MSC using a consumer‐grade polyimide tape as a precursor.^[^ ^28^ ^]^ Here, our approach is to pyrolyze the polyimide polymer and transform it into porous carbon that resembles graphene‐like carbon while precisely forming the microelectrode pattern using a high‐intensity laser.^[^ ^29^ ^]^ Instead of coating certain active materials and laser‐patterning the microelectrode, the drive renders the computer‐designed interdigitate pattern onto the active materials.^[^ ^30^ ^]^ This process is readily scalable and cost‐effective compared to the traditional MSC fabrication method.^[^ ^29^, ^30^ ^]^ The produced devices are extremely thin, lightweight, and ultra‐flexible in nature, making them the perfect choice in soft‐robotics applications. The laser Raman spectrum of the laser‐combusted carbon (Figure S1, Supporting Information) exhibits two dominant peaks, viz. i) *D* band (K‐point phonons of *A*_1g_ symmetry) at 1345 cm^−1^ induced due to the defects in GLC and ii) *G* band at 1577 cm^−1^ originating from *E*_2g_ phonons of C sp^2^ atoms, respectively.^[^ ^28^ ^]^ The field‐emission scanning electron microscopy (FE‐SEM) micrographs at various magnification scales and corresponding elemental mapping analysis for the laser‐combusted GLC are presented in Figure S2 (Supporting Information). The laser‐exposed microelectrode clearly reveals the formation of sheet‐like crumpled nanostructures with high porosity. The pore size of the carbonized GLC is not uniform (varies from 1 to 5 µm), which might be attributed to the intense laser beam used for scribing the microelectrode. Figure S2 (Supporting Information) (I) represents the elemental map for the carbon (C) in the laser‐combusted electrode, indicating the homogeneous distribution of *C* in the electrode. These studies confirmed the formation of graphene‐like carbon from the laser combustion technique.

### 2.1 Energy Storage Performances of Laser‐Combusted Graphene‐Like Carbon Micro‐Supercapacitor

**Figure 2:**
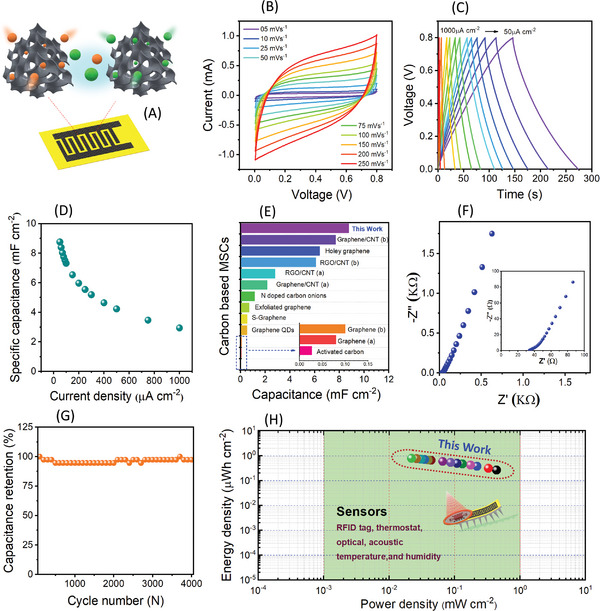
Electrochemical performance metrics analysis of GLC‐MSC using PVA/H_2_SO_4_ . A) Schematic of GLC‐MSC device, B) CV curves of GLC‐MSC measured at various sweep rates, C) GCD curves of GLC‐MSC at various applied current densities. D) The plot of specific capacitance versus current density. E) Comparing the areal capacitance (C/A) of this study to earlier published carbon‐based MSCs, indicating the GLC‐MSC has a substantially higher (C/A) than reported MSCs. F) Nyquist plot of GLC‐MSC device, inset of Figure (F) shows the enlarged portion of the high‐frequency region. G) Cycling performance of the GLC‐MSC (measured at a constant charge/discharge current density of  750µA cm^−2^). H) Ragone plot of GLC‐MSC with estimated practical micro‐sensory applications.

**Figure** **2**A illustrates the schematic representation of GLC‐MSC examined in the H_2_SO_4_/PVA gel electrolyte. As shown in Figure 2B, the GLC‐MSC device exhibits symmetric quasi‐rectangular shaped cyclic voltammetry (CV) curves by scanning from 0.0 to 0.8 V at sweep rates from 5 to 250 mV s^−1^, confirming that the mechanism of charge storage is purely due to the electric double‐layer (EDL) behavior of the GLC electrode. With an increase in sweep rate from 5 to 250 mV s^−1^, the current range of the CV curves increases linearly, deprived of any distortion in the rectangular shape that highlights the fast charge propagation of the GLC‐MSC.^[^ ^30^ ^]^ Even at a higher sweep rate of 1000 mV s^−1^, the CV curve remains rectangular in shape, indicating the high‐power capability of this GLC‐MSC (see Figure S3, Supporting Information). Figure S4 (Supporting Information) represents the continuous galvanostatic charge–discharge (GCD) curves of the GLC‐MSC device recorded at an applied current of 100 µA cm^−2^ that shows the symmetric triangular curves with a small voltage drop (0.01 V), implying the ideal capacitive behavior.^[^ ^31^ ^]^ Furthermore, the GCD curves of the GLC‐MSC device measured at various current ranges (from 50 to 1000 µA cm^−2^) are provided in Figure 2C, which shows the fast charging and discharging curves of the device. The areal capacitance calculated from the GCD curves at various current densities is shown in Figure 2D, where the device delivered a maximum capacitance of 8.76 mF cm^−2^ at a constant discharge current of 50 µA cm^−2^. With the increase in the current to 1000 µA (20‐fold), the GLC‐MSC still possesses an areal capacitance of 2.93 mF cm^−2^, implying a better rate capability. Figure 2E highlights the superior device capacitance properties of the GLC‐MSC compared to reported carbon‐based MSCs (see Table S1, Supporting Information). The Nyquist plot of the GLC‐MSC device (given in Figure 2F) and the inset shows the enlarged portion of the higher‐frequency region, revealing the equivalent series resistance (ESR) value ≈35 Ω, signifying negligible interfacial impedance issues in the GLC electrode.^[^ ^26^ ^]^ In addition, the GLC‐MSC device sustained ≈97.3% capacitance retention even after 4000 charge–discharge cycles at a constant current density of 750 µA cm^−2^ (see Figure 2G), indicating excellent electrochemical stability. Figure 2H shows the Ragone plot of the fabricated GLC‐MSC with estimated practical micro‐sensory applications, such as thermostat, optical, acoustic, temperature, humidity, etc.^[^ ^25^, ^26^, ^27^ ^]^

### 2.2 Performance Evaluation of the GLC‐MSCs at Various Actuation Conditions

**Figure 3:**
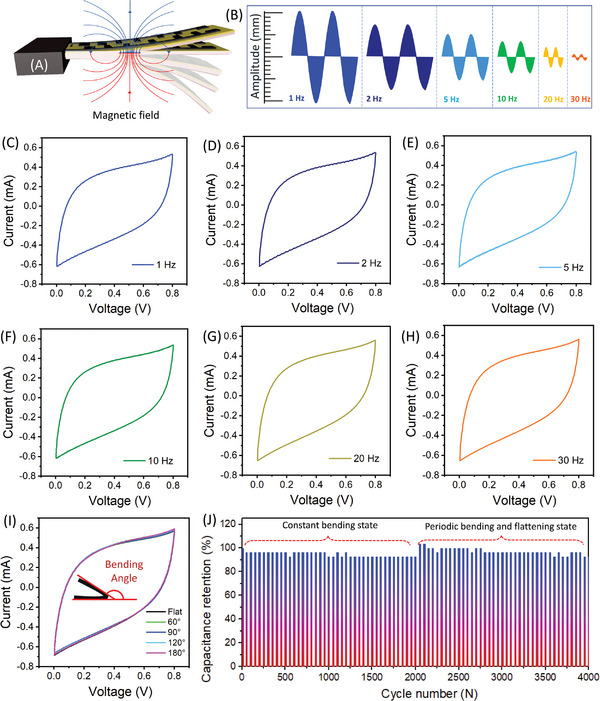
GLC‐MSC operation test in harsh conditions. A) Schematic of GLC‐MSC loaded magnetic actuator in the presence of the external magnetic field. B) Maximum actuation amplitude (in mm) of GLC‐MSC loaded magnetic actuator at the various actuation frequencies (1–30 Hz). C–H) CV curves of the GLC‐MSC under periodic bending frequencies (1–30 Hz), indicating the consistency in the performance of GLC‐MSC. I) CV curves of GLC‐MSC measured at various bending angles (0°–180°). J) Long‐term cycling stability test of GLC‐MSC under constant bending state as well as periodic bending and flattening state.

To demonstrate the uninterrupted operation aptitude of the GLC‐MSC in various dynamic environments, including bending and actuating at multiple frequencies (1–30 Hz) conditions. In this experiment, the prepared GLC‐MSC was applied on the surface of the thin magnetic PDMS actuator (dimension 40 mm × 12 mm, thickness ≈2 mm) and evaluated its charge storage characteristics under periodic actuation (1–30 Hz). The schematic model of the soft magnetic actuator with an onboard GLC‐MSC is shown in **Figure** **3**A. Here, the magnetic actuator undergoes a series of bending cycles driven by periodically switching the magnetic field. The maximum actuation amplitude (displacement in mm) of the GLC‐MSC loaded magnetic actuator at the various actuation frequencies (1–30 Hz) is provided in Figure 2B. Here, the amplitude of the actuator decreases gradually with an increase in the operating frequency (1–30 Hz), and this amplitude is almost lost above 20 Hz (see Figure 2B), respectively. (A detailed fabrication method of a magnetic actuator and experimental model is provided in Sections S1.3 and S1.4 and Figures S5 and S6, Supporting Information). Figure S7 (Supporting Information) represents the CV curve (at 100 mV s^−1^ sweep rate) of the GLC‐MSC recorded under a steady state (without actuation), implying the smooth and rectangular CV curves of the device. Similar to this, CV analysis of the GLC‐MSC device at a variety of actuation frequencies was also investigated. As expected, the GLC‐MSC device preserved its rectangular shape CV curve without any distortion within the actuation frequency range of 1–30 Hz, as seen in Figure 3C–H. At the lower frequency range (1–5 Hz) a very minute spike was observed in the CV curves, which might be attributed to the higher amplitude (±10–5 mm) actuation of the device. However, these spikes are vanishing slowly at the higher frequency range (above 5 Hz), as evident in Figure 3 (Movie S1, Supporting Information). Similar to this CD curve at an applied current of 500 µA was also recorded, indicating stable and smooth curves under the same circumstance of the magnetic field strength of ≈100 mT and actuation frequency of 5 Hz (see Figure S8 and Movie S2, Supporting Information). Likewise, the CV analysis at numerous pre‐deformation states (with bending angles of 0°–180°) was also performed that showed a similar type of CV curve with no notable variations, as seen in Figure 3I. Figure 3J shows the long‐term cycling stability test of the GLC‐MSC device, performed under constant bending, as well as periodic bending and flattening state over continuous 4000 charge–discharge cycles at an applied current of 750 µA, respectively. First, we examined the electrochemical stability (initial 2000 cycles) of the GLC‐MSC at constant bending conditions at ≈180°, ensuring that they maintained consistent functionality under static conditions. Following this, we subjected the MSC device to periodic bending and flattening state (at a controlled speed of 2 mm/s until the MSC reaches a bending angle of ≈180°). This bending process was designed to mimic the deformations experienced by soft robots during their operational cycles. Impressively, the GLC‐MSC showcased remarkable cycling stability over 4000 cycles with a capacitance retention of 92.8%. Here, we observed that during the initial cycles of periodic bending and flattening state, a slight evolution/increment in device capacitance (after 2000 cycles), which may be attributed to sudden changes in the strain and relaxation conditions experienced by the MSC device. However, this deviation from the original capacitance level was subsequently normalized and gradually reduced until the capacitance retention rate of 92.8%, as seen in Figure 3J. The capacitance retention value of GLC‐MSC is comparable to that of previously reported flexible MSC devices manufactured utilizing various electrode materials and fabrication methods, as seen in Table S2 (Supporting Information). Overall these investigations reveal that the fabricated GLC‐MSC device shows high‐frequency bending and deformation aptitude, minimizing the potential for failure or internal short circuits during harsh conditions.

### 2.3 Evaluating the Performance of Integrated GLC‐MSC Array for Empowering Sensory Systems

**Figure 4:**
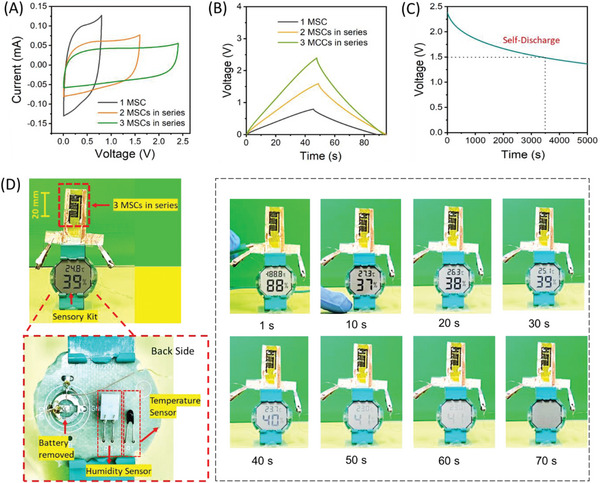
Evaluating the performance of GLC‐MSCs for empowering sensory systems. A–D) CV and CD curves of GLC‐MSCs (1–3 devices) connected in series. C) Self‐discharge analysis of GLC‐MSCs (3 devices in series). D) Real‐time photographs of micro‐sensory systems (that include a low‐power temperature sensor, humidity sensor, an electronic display, data processor, etc.), empowering with GLC‐MSC devices at various time periods (1–70 s).

To meet the voltage or power rating of micro‐sensing systems, we fabricated the three GLC‐MSCs in sequence. Since, the operational voltage window (OVW) of a single MSC device is limited to 0.8 V, which is inadequate for powering the majority of sensory systems. Thus, by combining three GLC‐MSCs in series the working voltage can be extended up to 2.4 V as evident from the CV and CD curves shown in **Figure** **4**A,B. Here, the device dimension is shortened due to limited space, and the three GLC‐MSC (in series) are arranged so that they fit within the dimensions of the soft‐robot. The complete electrochemical analysis (CV and CD curves) for a single GLC‐MSC is provided in Figure S9 (Supporting Information). Furthermore, the open circuit voltage (OCV) is a crucial parameter to characterize the self‐discharge process in the SCs. The GLC‐MSC cells (three MSCs in series) were charged up to 2.4 V and held at this voltage for 500 s (see Figure S10, Supporting Information). Subsequently, the GLC‐MSCs applied voltage was withdrawn, and monitored their OCV over 5000 s. Surprisingly the GLC‐MSC cells retain up to 1.5 V OCV during the 3500 s, and this was further reduced to 1.4 V only even after 5000 s, which is 58% of their initial OCV (see Figure 4C). This result clearly shows the superior charge‐holding capability of the GLC‐MSC devices. To demonstrate the real‐time applicability of our GLC‐MSCs devices, we charged them to 2.4 using a constant current of 2 µA and subsequently discharged them through the commercially available low‐power sensor kit (which contains four components such as a low‐power temperature sensor, a humidity sensor, as well as electronic display, and a data processor). As expected, the energy and power rating of the fabricated GLC‐MSC array is sufficient to power the moisture and temperature sensors and micro‐display over ≈50–60 s on a single charge, as seen in Figure 4D and Movie S3 (Supporting Information). These results indicate the reliability of GCL‐MSC as a on‐chip power source for micro‐sensing systems in soft robots.

### 2.4 Locomotion and Performance Evaluation of Fully Integrated Magnetic Soft‐Robot

**Figure 5:**
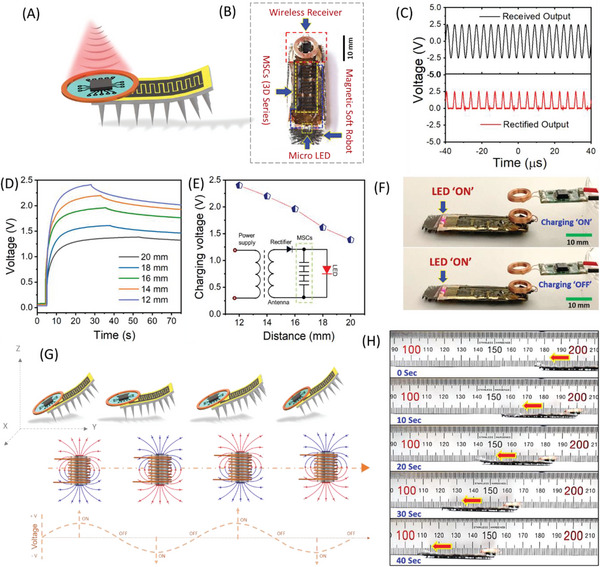
Demonstration of the magnetic soft‐robot with onboard GLC‐MSC and wireless charging system. A,B) Schematic and real‐time image of magnetic soft‐robot with onboard GLC‐MSC and wireless charging system. C) Output voltage of the fabricated wireless circuit. D) Wireless charging and self‐discharge curves of GLC‐MSCs according to the transmission distance. E) Wireless charging voltage at various transmission distances (from 12 to 20 mm), inset of Figure (E) shows the circuit diagram of the fully integrated magnetic soft‐robot with wireless charging and storage system. F) Photographs of fully integrated soft‐robot (top, charging state; bottom, discharging state with LED on‐state). G) Schematic illustration of the flap‐wave locomotion mode under the presence of a periodic magnetic field in the *Y‐Z* plane. H) Real‐time images of fully integrated magnetic soft‐robot locomotion in the flap‐wave mode.

Specifically, soft robots are very small in dimensions (from milli‐scale to micro‐scale) that contain multiple sensory and communication modules for real‐time data acquisition and transmission.^[^ ^32^ ^]^ Importantly, designing the lightweight and compact onboard power source to drive these sensory is still a big challenge.^[^ ^32^, ^33^, ^34^ ^]^ In this current work, we have designed fully integrated soft robots, which are composed of a wirelessly rechargeable GLC‐MSC device integrated with a rectifier circuit and receiver antenna. Importantly, here the weight of the fabricated GLC‐MSC cells is ≈50 milligrams, which is only 5.2% of the total weight of a soft robot (950 milligrams), respectively. The schematic illustration and real‐time photograph of the designed fully integrated multilegged soft robot are provided in **Figure** **5**A,B. The upper module of the soft robot includes the wireless power receiver (WPR) unit and GLC‐MSCs, which repeatedly store and use electric energy. While the lower module contains a multilegged soft robot. The designed prototype's overall dimension is comparable to a fingertip (≈12 mmx 40 mm).

Further, the wireless power transmission (WPT) functionalities are essential to improve the convenience of soft robotics. Figure 5C depicts the rectifying features of the WPT unit. The antenna (black lines in Figure 5C) was used to receive power inductively in this wireless circuit. The received power signal by the antenna as an alternative current (AC) was converted into the rectified output (red line in Figure 5C) with the help of a high‐frequency half‐wave rectifier and storage in the MSC cells. This wireless power transmission rate is dependent on the distance between the transmission and receiving antennas, and Figure 5D shows the wireless charging/ self‐discharging curves of GLC‐MSC cells at various transmission distances (12 to 20 mm), respectively. The MSCs were charged up to their full capacity (2.4 V) at a transmission distance of 12 mm within ≈21 s, indicating that 12 mm is the optimal distance for efficient charging. When the distance increased above this threshold, the charging voltage degraded with respect to the increase in distance (see Figure 5D). Figure 5E summarizes the dependence of this wireless charging/ self‐discharging characteristic on the transmission distance (from 12 to 20 mm). The circuit diagram of this wirelessly rechargeable MSC system, which includes an antenna, rectifier, GLC‐MSCs, and LED light, is provided in the inset of Figure 5E. Here, the LED is integrated as an indicator to demonstrate the wireless operation of a fully integrated soft robot. When this soft robot was powered wirelessly, the embedded LED displayed the state of wireless charging and discharging of MSC devices (see Figure 5F). As seen in the inset of Movie S4 (Supporting Information), wireless charging of these MSCs (for ≈21 s) kept the LED emission in the “on” state for almost ≈15 s.

To evaluate the locomotion capability of a fully integrated soft robot, we perform the operation of the robot under the circumstance of the same magnetic field strength (100 mT), and actuation frequency of 1 Hz. The schematic illustration and real‐time images of a fully integrated soft robot in the flap‐wave locomotion mode under the application of an external magnetic field are shown in Figure 5G,H. When the electromagnet is turned “ON,” the inflection angle of the soft robot decreases due to the magnetic torque and gradient force, and the robot travels forward in a step‐by‐step procedure until it hits the ground (basic theories about robot locomotion dynamic control can be found in Section S1.5 and Figure S11, Supporting Information). When the external magnetic field is turned “OFF,” the robot returns to its pre‐deformation deflection condition. With this repeated process and predefined conditions, the robot travels a 75 mm distance in ≈45 s in the *Y‐Z* plane in the full integration state, as seen in Figure 5H and Movie S5 (Supporting Information). The relative locomotion speed (≈0.041 body length/ s) and body mass (950 mg) of this fully integrated magnetic soft‐robot are comparable to state‐of‐art reported soft‐robots/actuators using various driving mechanisms, as seen in Table S3 (Supporting Information). Notably, all of these findings were achieved without sacrificing the GLC‐MSC's storage capability or their structural flexibility, indicating that the MSC could be a superior choice for next‐generation soft robotics applications.

## 3 Conclusion

In conclusion, we have demonstrated an untethered magnetic soft robot with an integrated micro‐supercapacitor that can charge wirelessly. The idea of using direct laser combusted GLC‐MSC shows superior areal capacitance (8.76 mF cm^−2^), promising energy density (0.778 µWh cm^−2^), and an excellent life cycle, capable of empowering the various senses. It is noteworthy that the fabricated MSC has several key advantages, such as they are robust, ultra‐lightweight (only 5.2% of the soft robot's weight), and capable of operating at high bending frequencies (1–30 Hz), and bending (0–180°) conditions without sacrificing charge storage performance. Additionally, the fully integrated magnetic‐soft robot with a wireless charging system and GLC‐MSC displayed an outstanding locomotion attitude and charged wirelessly (up to 2.4 V within 21 sec), making them an excellent onboard power source for soft robotic applications.

## 4 Experimental Section

### Fabrication of Laser‐Induced Porous Graphene‐Like Carbon Micro‐Supercapacitor

The porous graphene‐like carbon interdigital electrodes were fabricated using a commercial polyimide source via direct laser combustion technique (NEJE MASTER 2, 700 mW, laser wavelength 450 nm). First, the commercial polyimide tape (Kapton, thickness ≈65 µm) was applied to the top surface of the as‐prepared soft‐actuator/robot and laser‐irradiated (at optimized laser power and speed set to 40% and 3 mS (for 3 repetitive cycles)) to get highly porous graphene‐like carbon microelectrodes. The distance between the laser source and the substrate (*Z*‐axis) was fixed at 5 cm throughout this process. The dimension of each microelectrode was maintained with the NEJE controller software.

### Preparation of PVA‐H2SO4 Gel Electrolyte

The polymer gel electrolyte (PVA/H_2_SO_4_) was prepared by following the earlier reported method.^[^ ^35^ ^]^ Briefly, 1 g of PVA was dissolved in the 10 mL of DI water using a magnetic stirrer kept at 80 °C temperature to get a homogeneous solution. Afterward, an appropriate quantity of H_2_SO_4_ acid was added dropwise and allowed to magnetic stir until they got clear and translucent gel. Lastly, the obtained PVA/H_2_SO_4_ gel allowed cooling at room temperature and fabricated a symmetric micro‐supercapacitors device by casting the prepared PVA/H_2_SO_4_ gel electrolyte.

### Fabrication of Soft‐Magnetic Actuator and Multi‐Leg Soft Robot

The soft magnetic actuator and multi‐leg soft robot were fabricated via a ferromagnetic particle‐assisted molding approach (the detailed preparation method is provided in Section S1.3, Supporting Information).

### Electrochemical Analysis

The specific device capacitance (*C*_sp_) of the GLC‐MSC was calculated from the CV and CD analysis using the relation:^[^ ^26^ ^]^

(1) $$C_{\left(\mathrm{Areal}\right)}=\int I\mathrm{dV}/\left(s\times \Delta V\times A\right)$$

(2) $$C_{\left(\mathrm{Areal}\right)}=\left(I\times \Delta t\right)/\left(\Delta V\times A\right)$$

Here “*C*_(Areal)_” is the specific areal capacitance (F cm^−2^) of GLC‐MSC, “I” is the current (A), “s” is the scan rate (mV s^−1^), “ΔV” is the voltage window (V), “∆t” is the discharge time (s) and “A” is the active area of the electrode (cm^−2^). The energy (E) and power (P) density of the GLC‐MSC are calculated using the relations 4:

(3) $$E=0.5\times C_{\left(\mathrm{Areal}\right)}\times \Delta V^{2}$$

(4) P=E/Δt

## Conflict of Interest

The authors declare no conflict of interest.

## Supporting information

Supporting Information

Supplemental Movie 1

Supplemental Movie 2

Supplemental Movie 3

Supplemental Movie 4

Supplemental Movie 5

## Data Availability

The data that support the findings of this study are available from the corresponding author upon reasonable request.
