# Supplementary material for: Untethered Magnetic Soft Robot with Ultra‐Flexible Wirelessly Rechargeable Micro‐Supercapacitor as an Onboard Power Source
Source: Adv Sci (Weinh). 2023 Aug 6;10(28):2303918. doi: 10.1002/advs.202303918 (PMC10558651; doi:10.1002/advs.202303918)
Supplement: Supplementary file 1 — Supporting Information [file ADVS-10-2303918-s005.pdf]

ADVANCED SCIENCE
Open Access

Supporting Information

for *Adv. Sci.*, DOI 10.1002/advs.202303918

Untethered Magnetic Soft Robot with Ultra-Flexible Wirelessly Rechargeable Micro-Supercapacitor as an Onboard Power Source

*Swapnil Shital Nardekar and Sang-Jae Kim**

Untethered Magnetic Soft Robot with Ultra-Flexible Wirelessly Rechargeable Micro-Supercapacitor as an Onboard Power Source

Swapnil Shital Nardekar[1], and Sang -Jae Kim[1,2,3*]

[1]Nanomaterials & System Lab, Major of Mechatronics Engineering, Faculty of Applied Energy System, Jeju National University, Jeju 63243, Republic of Korea.

[2]Nanomaterials & System Lab, Major of Mechanical System Engineering, College of Engineering, Jeju National University, Jeju 63243, Republic of Korea.

[3]Research Institute of New Energy Industry (RINEI), Jeju National University, Jeju 63243, Republic of Korea.

*Corresponding author Email: kimsangj@jejunu.ac.kr

Supplementary Materials

Fig. S1. Raman structure of laser combusted GLC-electrode.

Fig. S2. FE-SEM micrographs of laser combusted GLC-electrode.

Fig. S3. CV curves of GLC-MSC at higher sweep rate.

Fig. S4. Continuous CD curves of GLC-MSC.

Fig. S5. Detailed fabrication procedure of the multi-legged magnetic soft robot.

Fig. S6. Experimental set-up used for performance evaluation of the GLC-MSC devices at various actuation conditions.

Fig. S7. CV curves of GLC-MSC measured under a steady state.

Fig. S8. CD curves of GLC-MSC measured recorded under an actuation frequency (5-Hz).

Fig. S9. Electrochemical analysis of GLC-MSC device.

Fig. S10. Chrono amperometry charging profile of GLC-MSC.

Fig. S11. Locomotion analysis of fully integrated magnetic soft-robot.

Table S1. Comparison of areal-specific capacitance of GLC-MSC with reported carbon based MSCs.

Table S2. Comparison of capacitance retention (%) of GLC-MSC with reported MSC devices based on the various fabrication mathods and bendinding conditions.

Table S3. Comparison of locomotion speed and mass data of various soft-robot or actuator with current work.

Movie S1. Cyclic voltammetry curves recorded under actuation frequency (5 Hz).

Movie S2. Galvanostatic charge discharge curves recorded under actuation frequency (5 Hz).

Movie S3. Powering the micro-sensory system using GLC-MSC devices

Movie S4. Wireless charging of onboard GLC-MSCs array and detection through powering the red LED.

Movie S5. Locomotion analysis of a fully integrated magnetic soft robot (at 1Hz).

S1. Experimental Section

S1.1 Materials

The starting precursor materials such as nickel micropowder, polydimethylsiloxane (PDMS), phosphoric acid (H_3PO_4), and polyvinyl alcohol (PVA) were purchased from Daejung metals and chemicals, South Korea. The iron microparticles are obtained by manually grinding an iron rod using a disc grinder machine (with an approximate size ranging from 500 to 1000 μm). The commercial grade polyimide tape and electronic components were perchased from eleparts korea. All the materials used in this work are of research grade and used without further purification.

S1.2 Instrumentation

The laser Raman spectroscopy of the GLC-MSC electrode was carried out with the help of the Lab Ram HR Evolution Raman spectrometer (Horiba Jobin-Yvon, France, at a laser excitation source of wavelength 514 nm). The morphological examination of GLC-MSC was carried out using field-emission scanning electron microscopy (TESCAN, MIRA3) at various magnifications in conjunction with energy dispersive X-ray (EDS) spectroscopy. Bending testing of the GLC-MSC was carried out using bending tester equipment (JUNIL-JIBT-200). The electrochemical characterization was carried out with the help of an Autolab PGSTAT302N electrochemical workstation. The magnetic field strength and electromagnetic polarity were adjusted using a function generator (FG-1883 5MHz DDS function generator) and a power amplifier (Labworks Inc. pa-133). The oscilloscope was used to measure the wireless power output (Tektronix MD3034).

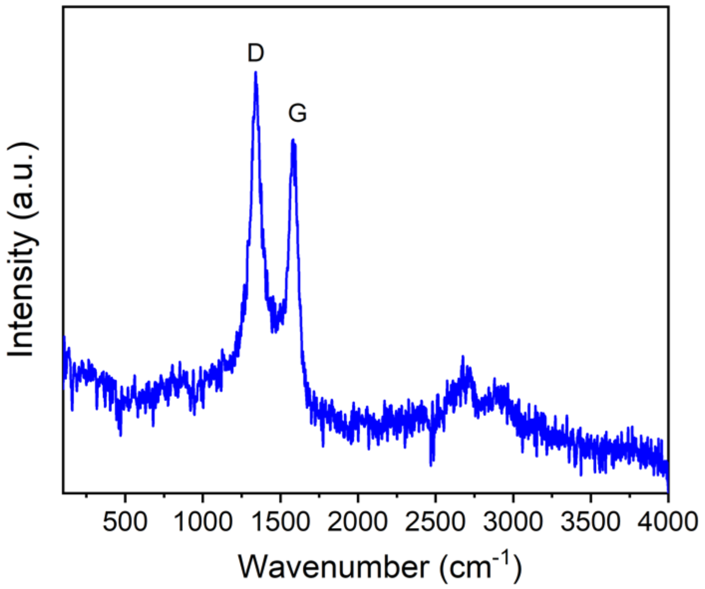

Figure S1: Raman structure of laser combusted GLC-electrode.

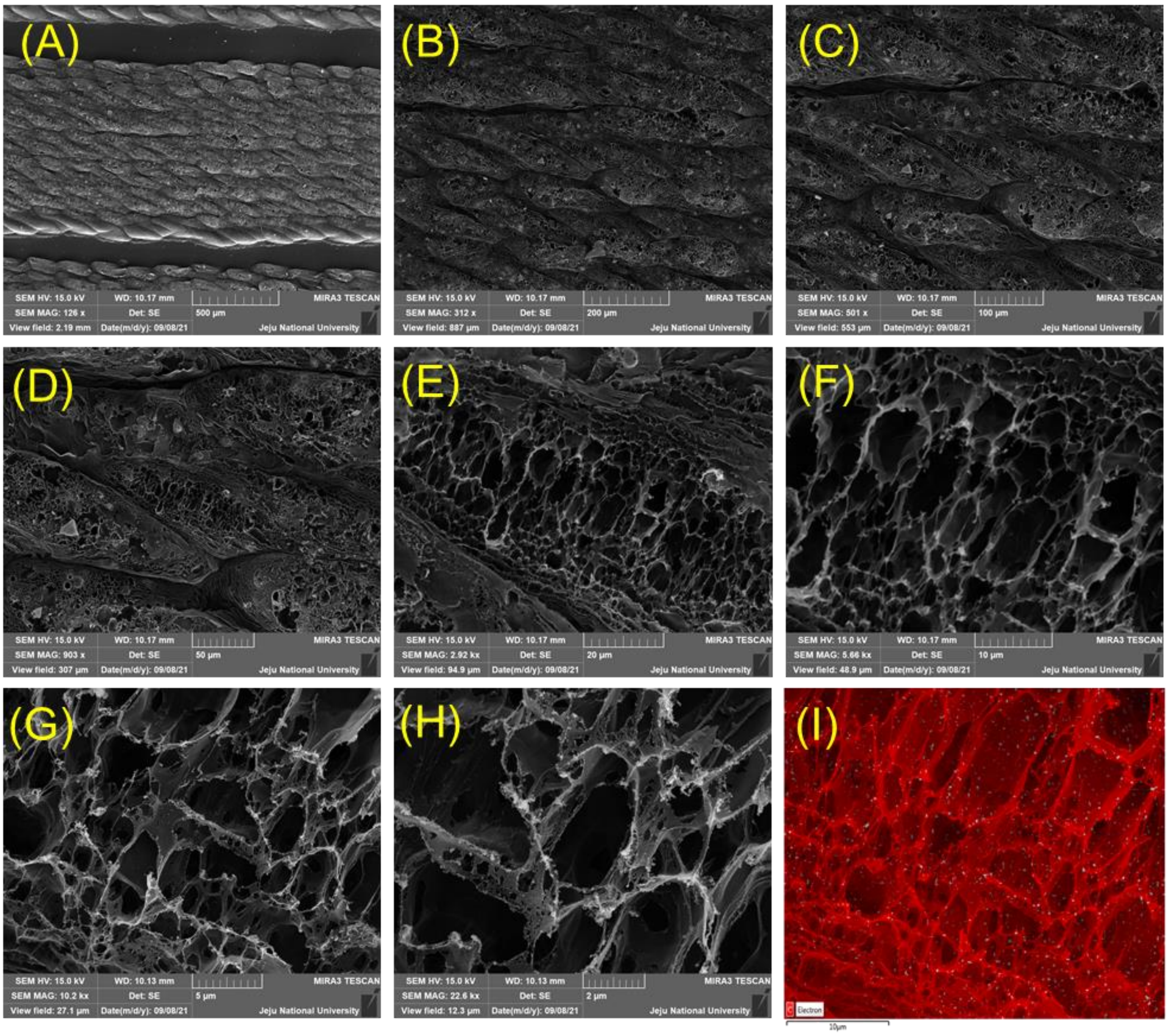

Figure S2. (A-H) FE-SEM micrographs of laser combusted GLC-electrode at various magnification scales, (I) elemental mapping image for the carbon present in the GLC-electrode.

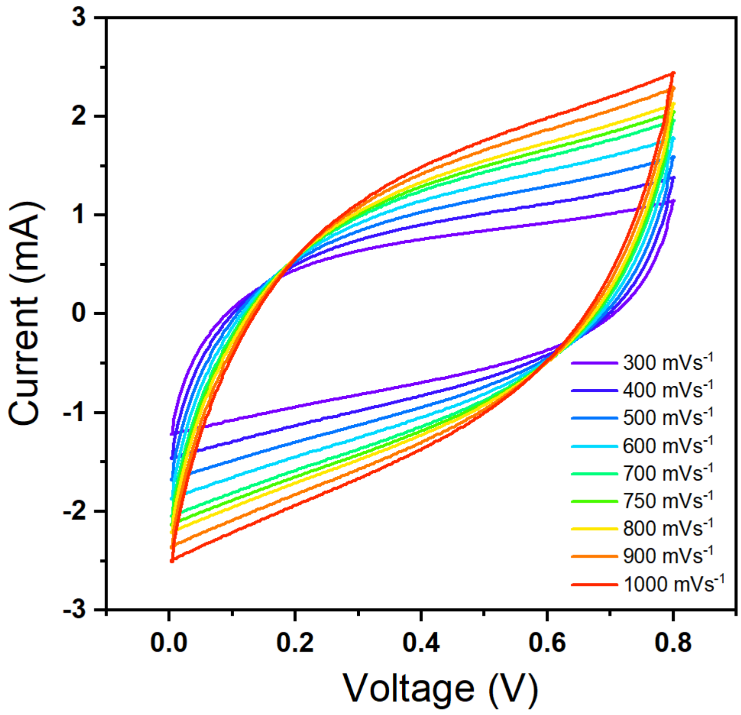

Figure S3. CV curves of GLC-MSC at higher sweep rate ranging from 300-1000 mVs^{-1}.

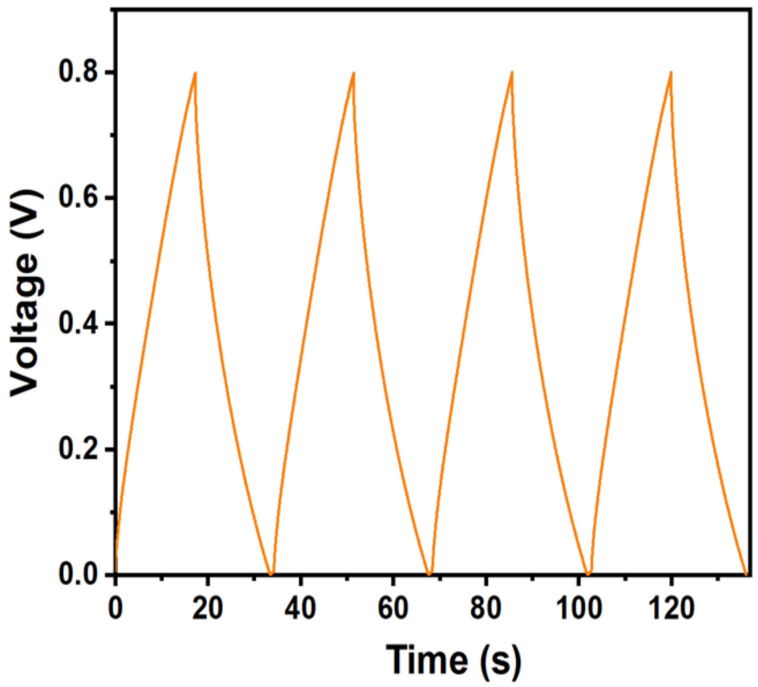

Figure S4. Continuous CD curves measured at an applied current density of 100 μA cm^{-2}

S1.3 Fabrication of soft-magnetic actuator and multi-leg soft robot

The soft magnetic actuator was fabricated via a modified ferromagnetic particle-assisted molding approach[1]. Firstly, a polydimethylsiloxane (PDMS) pre-polymer (1 gm) nickel, and iron microparticles in a volume ratio of 1:0.5:0.5 were uniformly mixed for 10 min using a magnetic stirrer. Then, the desired amount of curing agents was added to the earlier mixture and mixed for 10 minutes to ensure the homogeneous distribution of magnetic particles. The well-mixed magnetic solution was transferred into a petri dish and cured in the vacuum oven at 60 °C for 2 hrs. Then, the cured magnetic PDMS composite film was cut into desired shapes and used as a soft-magnetic substrate with the loading of the micro-supercapacitor power source.

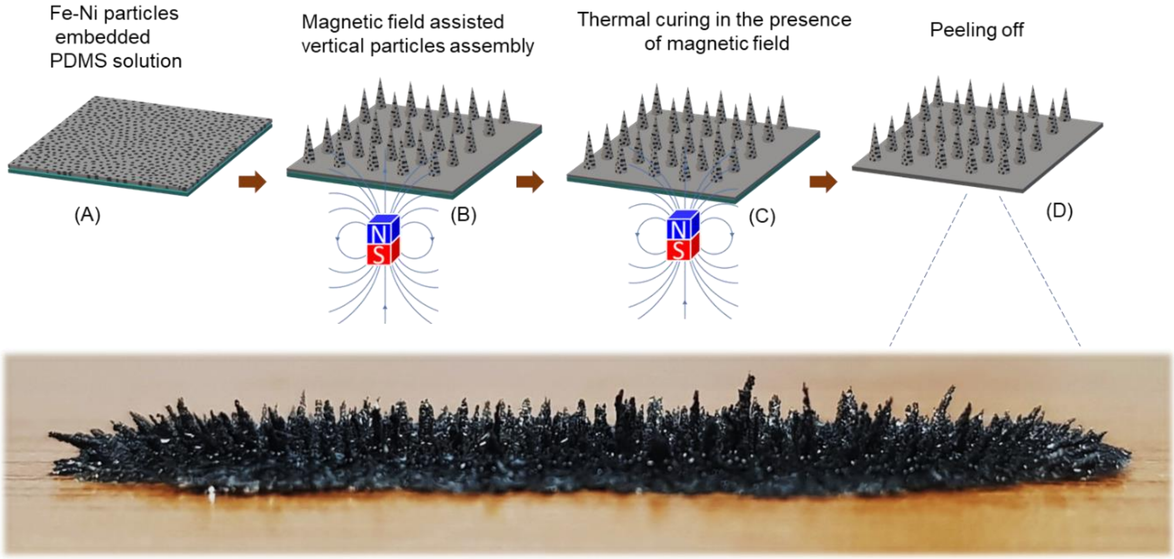

Figure S5: Detailed fabrication procedure of the multi-legged magnetic soft robot.

Similarly, the magnetic multi-leg robot was fabricated by dispersing the desired amount of a prepared magnetic solution on the acrylic plate through the drop-casting process. Afterwards, a permanent niobium magnet was applied underneath the plate to form ordered micro-feet like structure. The magnetic field intensity of ~540 mT was

maintained by keeping the distance between the niobium magnet and the acrylic plate constant (0.5 cm). After developing the micro legs on the acrylic plate, the plate was then transferred vacuum oven for thermal curing for 2 h at 60 °C[1,2]. After solidification, we pulled the magnetic PDMS away from the underlying substrate and trimmed it to the proper size (dimension 40 × 12 mm, thickness ~ 2 mm) to create our multi-legged soft robot.

S1.4 Performance evaluation of the GLC-MSC devices at various actuation conditions

In this experiment, the prepared GLC-MSC device was applied on the surface of the thin magnetic PDMS actuator and evaluated its charge storage characteristics under periodic actuation (1 to 30 Hz). The schematic model of the soft magnetic actuator with an onboard GLC-MSC is shown in Fig. S6.

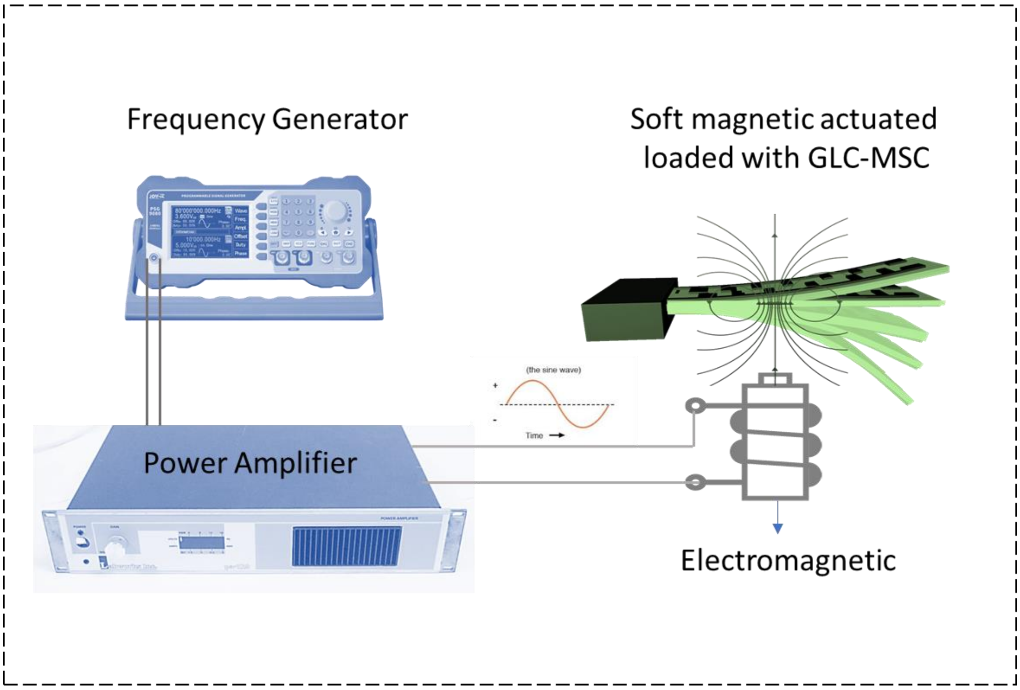

Figure S6. Experimental set-up used for performance evaluation of the GLC-MSC devices at various actuation conditions.

The magnetic actuator is subjected to a series of bending cycles triggered by switching the magnetic field ON and OFF. The frequency generator and power amplifier are used to regulate the periodic functioning of the electromagnet (at various frequency ranges). The created magnetic field draws the magnetic actuator towards its head (in ON state) and returns to its original location periodically (in OFF condition) due to its elastic nature. The amplitude of the actuator drops progressively as the working frequency increases (1 to 30 Hz), and it is nearly eliminated above 20 Hz. During this procedure, the performance of MSC devices was assessed using CV and CD, respectively.

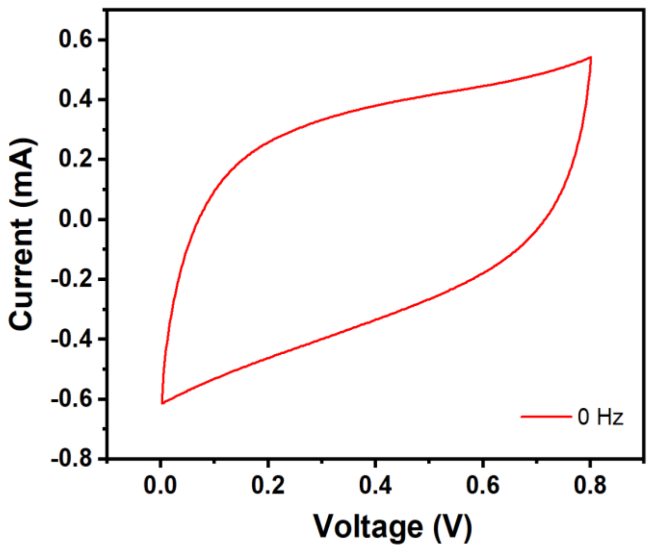

Figure S7. CV curves of GLC-MSC measured at 100 mVs^{-1} sweep rate recorded under a steady state (without actuation)

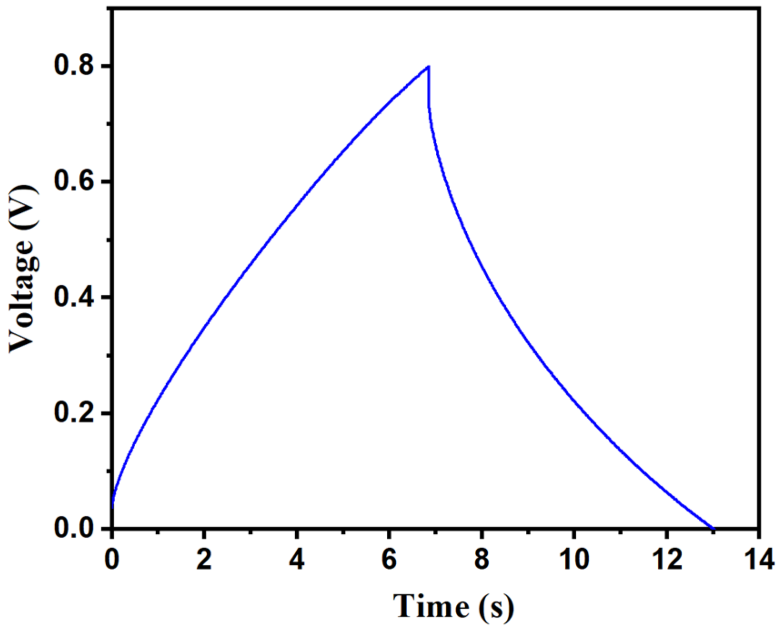

Figure S8. CD curves of GLC-MSC measured at 500μA current recorded under an actuation frequency (5-Hz)

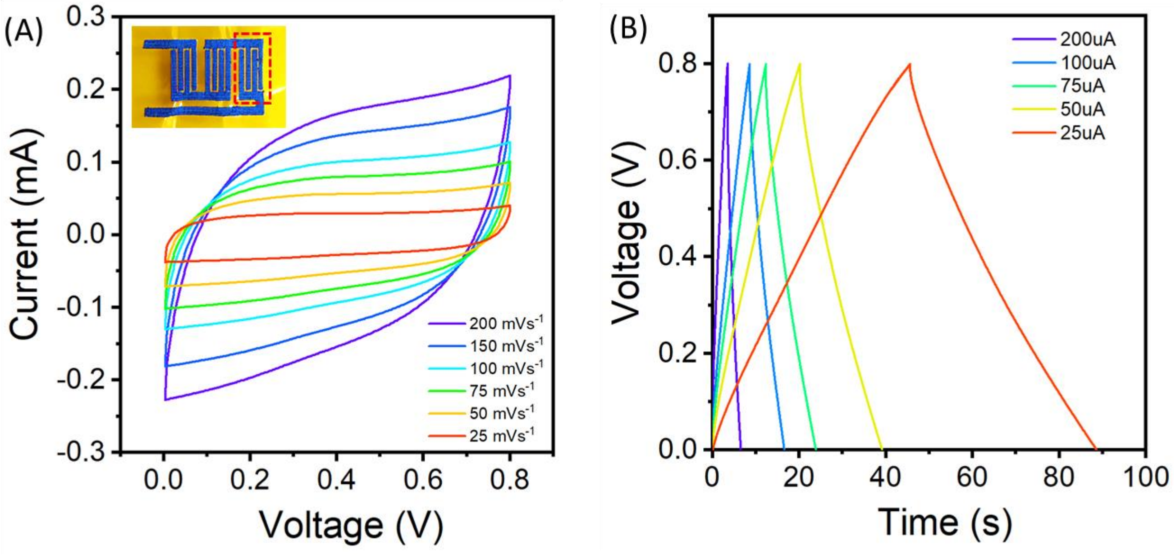

Figure S9. Electrochemical analysis of GLC-MSC device, (A) CV curves at various sweep rates (25-100 mVs^{-1}), (B) CD curves at various current (25-200 μA)

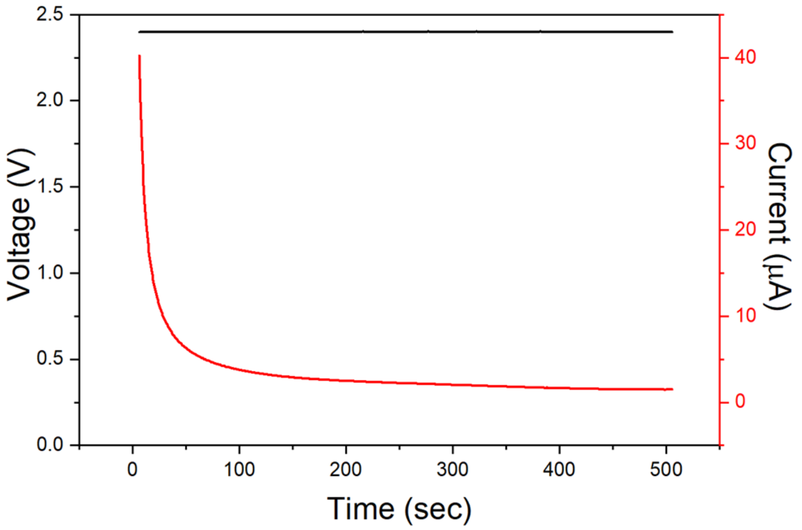

Figure S10. Chrono amperometry charging profile of GLC-MSC (3D in series combination)

S1.5 Locomotion analysis of fully integrated magnetic soft robot

The untethered, fully integrated soft-scale soft robot demonstrates the flap-wave locomotion mode when the alternative switching of the magnetic field is applied in the Y-Z plane, as shown in Fig. S11 (see Movie S5).

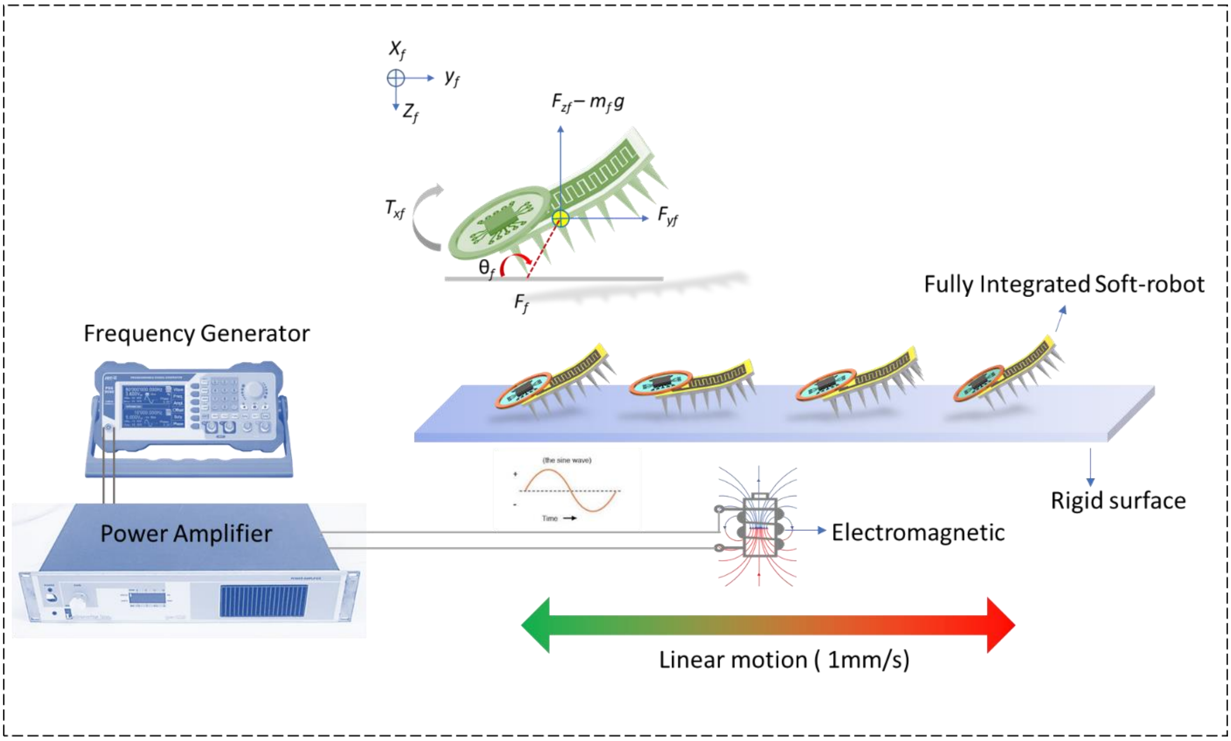

Figure S11. Locomotion analysis of fully integrated magnetic soft-robot.

At the initial state, when the electromagnet is located underneath the soft robot in the 'OFF' condition, the robot with the pre-deformation angle maintains the inflection posture. When the electromagnet is turned 'ON', the inflection angle of the robot lowers under the influence of magnetic torque and gradient force, respectively. The robot's front feet are lowered to align the magnetic induction line, and the robot is pulled forward to the location of the strongest magnetic field. The robot advances step by step until its legs

are completely on the ground. After the magnetic field turned ‘OFF’, the robot returns to its starting state under the pre-deformation deflection. Furthermore, soft robots’ flap-wave locomotion on the 2D plane can be expressed through the dynamic model as follows[1,2]:

$$m_f \ \ddot{y}_f = F_{yf} - F_f \text{ --------------------------------------(5)}$$

$$m_f \ddot{z}_f = F_{zf} - m_f g \text{ --------------------------------------(6)}$$

$$J \ \ddot{\theta}_f = T_{xf} + F_f \, . \, r_f . \sin \theta_f \text{--------------------------------------(7)}$$

where $\ddot{y}_f$ and $\ddot{z}_f$ denotes the robot's acceleration along the y and z axes, respectively. The magnetic force experienced by the robot along the y and z axes is represented by F_{yf} and F_{zf}, respectively. The mass of the robot is m_f, and the acceleration of gravity is g. The sliding friction force is represented by F_f. J in the polar moment of inertia of the soft robot. Further, $\ddot{\theta}_f$ and θ_f denote orientation angle and angle angular acceleration, respectively. The distance between the robot's centre of mass (COM) and the ground-contacting point is indicated by r_f.

Table S1: Comparison of areal-specific capacitance of GLC-MSC with reported carbon based MSCs.

No.	Materials	Method of Fabrication	Voltage Window	Areal specific capacitance (mF cm^{-2})	Ref.
1.	Activated carbon film	Lithographic technique	0.6V	0.027	[3]
2.	Graphene	Plasma etching	1.0V	0.08	[3]
3.	Graphene	Inkjet printing	1.0V	0.1	[3]
4.	Graphene quantum dots	Electro-deposition dots	1.0V	0.5347	[3]
5.	S- Graphene	Plasma etching	1.0V	0.553	[3]
6.	Exfoliated Graphene Inkjet printing	Inkjet printing	1.0V	0.7	[3]
7.	N-doped carbon onions	Electrophoretic deposition	1.0V	1.16	[3]
8.	Graphene/CNT	Photolithography and chemical vapour deposition	1.0V	2.16	[3]
9.	RGO/CNT	Electrostatic spray deposition and photolithography lift-off methods	1.0V	2.8	[3]
10.	RGO/CNT	Electrostatic spray deposition	1.0V	6.1	[4]
11.	Holey Graphene	Atmospheric pulsed micro plasma-jet	1.0V	6.41	[5]
12.	Graphene/CNT	Screen printing	1.0V	7.7	[6]
**13.**	**Graphene like carbon**	**Laser combustion technique**	**0.8V**	**8.76**	**This Work**

Table S2: Comparison of capacitance retention (%) of GLC-MSC with reported MSC devices

No.	Electrode material	Fabrication method	Capacitance retention	Bending condition	Number of cycles	Ref.
1.	Graphene	Laser cutting	85%	Periodic bending (90°)	5,000	[7]
2.	CoO/CNT	Screen printing	99-101%	0 to 180°	1	[8]
3.	NiCoP@NiOOH//ZIF-C	Screen printing	100%	Periodic bending (90°) and flat state	500	[9]
4.	rGO/CNT	Ink jet printing	81.5%	Periodic bending (120°) and flat state	150	[10]
5.	BCN	Laser patterning	-	Constant bending (150 ^{o})	1,500	[11]
6.	Boron-doped diamond nanowire	Laser writing	96%	Constant bending (180 ^{o})	10,000	[12]
7.	PBO paper	Laser-induced graphene	85%	Constant bending (120 ^{o})	3,000	[13]
8.	$CNF@MnO_2$ $//CNF@Fe_2O_3$	Electrodeposition	96.3%	Flat, Bend, Stretch, Twist	100	[14]
9.	MnO_2/NBC/PEDOT	Pen lithography	96.6%	Periodic bending	500	[15]
**10.**	**Graphene like carbon**	**Laser combustion**	**92.2%**	**Periodic bending (180°) and flattening state**	**4,000**	**This work**

based on the various fabrication mathods and bendinding conditions.

Table S3: Comparison of locomotion speed and mass data of various soft-robot or actuator with current work.

**No.**	**Soft-robot / Actuator**	**Relative speed (body length/s)**	**Body Mass (g)**	**Ref.**
1.	Thermally activated soft-robot	0.02632	60	[16]
2.	Inchworm-inspired soft-robot	0.0184	63	[17]
3.	Fluidic soft-robot	0.07	900	[18]
4.	Artificial annelid robot	0.022	30	[19]
5.	Earthworm-like soft-robot	0.005	9	[20]
6.	Magnetic assembly of soft-robots	0.039	63	[21]
7.	Humidity driven walking robot	0.011	0.036	[22]
8.	Electro-actuated hydrogel walkers	0.003	0.052	[23]
9.	Bioinspired hygromorphic actuator	0.02	0.024	[24]
10.	Multilegged magnetic soft millirobot	0.029	0.0394	[1]
11.	IPMC actuator-based an underwater microrobot	0.138	17	[25]
12.	Underwater biomimetic microrobot	0.105	19	[26]
13.	Multifunctional underwater microrobot	0.024	10	[27]
**14.**	**Untethered Magnetic Soft Robot**	**0.041**	**0.95***	**This work**

*Only mass of soft-robot

References

[1] H. Lu, M. Zhang, Y. Yang, Q. Huang, T. Fukuda, Z. Wang, Y. Shen, *Nature Communications* **2018**, *9*, 3944.

[2] H. Lu, Y. Hong, Y. Yang, Z. Yang, Y. Shen, *Advanced Science* **2020**, *7*, 2000069.

[3] S. S. Nardekar, K. Krishnamoorthy, S. Manoharan, P. Pazhamalai, S.-J. Kim, *ACS Nano* **2022**, *16*, 3723.

[4] M. Beidaghi, C. Wang, *Advanced Functional Materials* **2012**, *22*, 4501.

[5] A.-K. Lu, H.-Y. Li, Y. Yu, *Journal of Materials Chemistry A* **2019**, *7*, 7852.

[6] J.-K. Chih, A. Jamaluddin, F. Chen, J.-K. Chang, C.-Y. Su, *Journal of Materials Chemistry A* **2019**, *7*, 12779.

[7] L. Zhang, D. DeArmond, N. T. Alvarez, R. Malik, N. Oslin, C. McConnell, P. Kwasi Adusei, Y.-Y. Hsieh, V. Shanov, L. Zhang, R. Malik, P. K. Adusei, Y. Hsieh, V. Shanov, D. DeArmond, N. T. Alvarez, N. Oslin, C. McConnell, *Small* **2017**, *13*, 1603114.

[8] Y. G. Zhu, Y. Wang, Y. Shi, J. I. Wong, H. Y. Yang, *Nano Energy* **2014**, *3*, 46.

[9] M. Qiu, P. Sun, G. Cui, Y. Tong, W. Mai, *ACS Nano* **2019**, *13*, 8246.

[10] W. Zhang, B. Li, R. Lv, H. Li, Y. Weng, W. Shen, F. Kang, Z. H. Huang, *Journal of Materials Chemistry A* **2023**, *11*, 9504.

[11] I. Karbhal, A. Basu, A. Patrike, M. V. Shelke, *Carbon* **2021**, *171*, 750.

[12] J. Ryl, D. Geng, S. Deshmukh, P. Jakobczyk, M. Ficek, J. Ryl, D. Geng, R.

Bogdanowicz, S. Deshmukh, P. Jakobczyk, M. Ficek, R. Bogdanowicz, *Advanced Functional Materials* **2022**, *32*, 2206097.

[13] M. Wang, J. Chen, K. Lu, Y. Ma, H. Li, J. Ye, *Electrochimica Acta* **2022**, *401*, 139490.

[14] Y. Yan, J. Yan, X. Gong, X. Tang, X. Xu, T. Meng, F. Bu, D. Cai, Z. Zhang, G. Nie, H. Zhang, *Chemical Engineering Journal* **2022**, *433*, 133580.

[15] H. R. Kim, J. H. Lee, S. K. Lee, Y. Chun, C. Park, J. H. Jin, H. U. Lee, S. W. Kim, *Journal of Cleaner Production* **2021**, *284*, 125449.

[16] N. Cheng, G. Ishigami, S. Hawthorne, H. Chen, M. Hansen, M. Telleria, R. Playter, K. Iagnemma, *Proceedings - IEEE International Conference on Robotics and Automation* **2010**, 5207.

[17] W. Wang, J. Y. Lee, H. Rodrigue, S. H. Song, W. S. Chu, S. H. Ahn, *Bioinspiration and Biomimetics* **2014**, *9*, 046006.

[18] C. D. Onal, D. Rus, *Bioinspiration & Biomimetics* **2013**, *8*, 026003.

[19] K. Jung, J. C. Koo, J. Do Nam, Y. K. Lee, H. R. Choi, *Bioinspiration & Biomimetics* **2007**, *2*, S42.

[20] B. Kim, M. G. Lee, Y. P. Lee, Y. Kim, G. Lee, *Sensors and Actuators A: Physical* **2006**, *125*, 429.

[21] S. W. Kwok, S. A. Morin, B. Mosadegh, J. H. So, R. F. Shepherd, R. V. Martinez, B. Smith, F. C. Simeone, A. A. Stokes, G. M. Whitesides, *Advanced Functional Materials* **2014**, *24*, 2180.

[22] Y. Ma, Y. Zhang, B. Wu, W. Sun, Z. Li, J. Sun, *Angewandte Chemie International Edition* **2011**, *50*, 6254.

[23] D. Morales, E. Palleau, M. D. Dickey, O. D. Velev, *Soft Matter* **2014**, *10*, 1337.

[24] S. W. Lee, J. H. Prosser, P. K. Purohit, D. Lee, *ACS Macro Letters* **2013**, *2*, 960.

[25] S. Guo, L. Shi, K. Asaka, *Proceedings of 2008 IEEE International Conference on Mechatronics and Automation, ICMA 2008* **2008**, 551.

[26] W. Zhang, S. Guo, K. Asaka, *Microsystem Technologies* **2007**, *13*, 883.

[27] L. Shi, S. Guo, K. Asaka, S. Mao, in *2010 IEEE International Conference on Nano/Molecular Medicine and Engineering*, IEEE, **2010**, pp. 1–6.
